# Combining IL-10 and Oncolytic Adenovirus Demonstrates Enhanced Antitumor Efficacy Through CD8^+^ T Cells

**DOI:** 10.3389/fimmu.2021.615089

**Published:** 2021-02-26

**Authors:** Duo Chen, Luyu Huang, Haiyu Zhou, Yuhui Zhang

**Affiliations:** ^1^ Department of Respiratory and Critical Care Medicine, Beijing Institute of Respiratory Medicine, Beijing Chao-Yang Hospital, Capital Medical University, Beijing, China; ^2^ Division of Thoracic Surgery, Guangdong Lung Cancer Institute, Guangdong Provincial People’s Hospital & Guangdong Academy of Medical Sciences, School of Medicine, South China University of Technology, Guangzhou, China

**Keywords:** cancer, oncolytic adenovirus, Ad-hTERT, IL-10, combination therapy, CD8+ T cells

## Abstract

Oncolytic viruses are of growing importance in cancer therapeutics since they combine direct oncolytic effect and the stimulation of antitumor immunity. Emerging evidences showed that the function of oncolytic viruses is dependent on immune response in tumor microenvironment, and the modulation of immunity could influence their efficacy. Here we combined the interleukin 10 (IL-10) and oncolytic adenovirus Ad-hTERT to treat lung cancer and explored the underlying mechanism under combination therapy. Lewis lung carcinoma (LLC) and B16F10 tumor-bearing immunocompetent C57BL/6 mice that received Ad-hTERT or IL-10 alone showed mild antitumor effect, while the combination therapy shrink tumor bulks and prolonged survival remarkably. In addition, IL-10 didn’t show direct influence on tumor cell viability or Ad-hTERT mediated tumor cell lysis *in vitro*. To further explore the influence of combination therapy mediated antitumor capacity, we eliminated CD8^+^ T, CD4^+^ T or natural killer (NK) cells in LLC and B16F10-bearing C57BL/6 mice, and found that CD8^+^ T cells were critical mediator in the combination therapy. The combination therapy induced intensive infiltration of CD8^+^ T cells in tumors, increased tumor-specific IFN-γ secretion by CD8^+^ T cells. The long-term tumor-specific immune memory induced by the combination therapy rejected rechallenge by respective tumor cell lines. This study demonstrated that the therapy combining IL-10 and Ad-hTERT augmented antitumor efficacy which was CD8^+^ T cells dependent. Our findings paved the way to combine cytokines and oncolytic viruses to enhance antitumor immunotherapy in treating cancer.

## Introduction

Oncolytic viruses are attracting growing interest in tumor treatment owning to their specific killing of tumor cells (1). Among the diverse oncolytic viruses evaluated in preclinical models and clinical stages, oncolytic adenovirus serotype 5 (Ad5) has been intensively investigated and showed potential efficacy in clinical trials (2–4). Apart from virus replication and direct killing of tumor cells, accumulating evidences support that the activation of antitumor immune response by oncolytic virus also contributes to tumor shrinking. And even the antitumor immunity stimulated by the tumor-injecting oncolytic viruses could inhibit the non-injected bulks. The released tumor antigens, and pathogen- and damage-associated molecular pattern molecules from lytic tumor cells could activate tumor-infiltrating antigen presenting cells which engulf, process and present antigens to effector immune cells, notably CD8^+^T lymphocytes (5, 6).

Oncolytic Ad5 is a promising antitumor alternative, and the combination of oncolytic Ad5 with immunotherapy has shown potential in eliminating tumors in pre-clinical models and clinical trials. The Ad5/3-Δ24-GM-CSF could release granulocyte-macrophage colony-stimulating factor (GM-CSF) that activates macrophages, leading to stable disease or minimal responses in half of patients with metastatic solid cancers **(** 7 **).** Ad-TD-nsIL-12 virus could express non-secreted IL-12 and showed CD8^+^ T-cell dependent antitumor efficacy, resulting in remarkable tumor regression in immunocompetent Syrian Hamster models with pancreatic cancer **(** 8 **).**

Telomeres are essential for genome stability and telomerase contributes to human carcinogenes through the maintenance of telomeres and cellular immortalization (9–11). The human telomerase reverse transcriptase (hTERT) plays a pivotal role in retaining the human telomerase activity, and hTERT promoter triggers hTERT gene expression selectively in tumor cells but is silent in most adult somatic cells (12). Ad-hTERT is an *E1*-deleting and replication defective oncolytic adenovirus, and hTERT promoter is used to promote the expression of E1 in telomerase-positive tumor cells, resulting in selective replication in tumor cells and tumor cell lysis. Ad-hTERT has been widely researched and showed promising tumor lysis potential (13–15). The tumor microenvironment comprises complicated immune factors, and the augmentation of antitumor immunity in the process of oncolytic virus mediated tumor regression is essential to exploit the maximum potential (16, 17).

Interleukin 10 (IL-10) is an immunoregulatory cytokine that can suppress aberrant inflammatory responses. However, the role of IL-10 in tumor development is controversial. According to some reports, IL-10 can be secreted by different types of cells, including numerous tumor cells (18, 19), and almost all leukocytes (20, 21). Furthermore, the serum level IL-10 in cancer patients are often increased, which often indicate poor prognosis (22). In the tumor microenvironment, Il-10 could inhibit macrophage cytokine synthesis and their antigen-presenting capacity **(** 23 **).** On the other hand, IL-10 showed stimulating roles in tumor-resident CD8^+^T cell through inducing cytotoxicity of CD8^+^ T cells and leads to an increase in the expression of IFN-γ in CD8^+^ T cells (24). Therefore, the different roles of IL-10 on tumor development are associated with different physiopathological states and the local environment.

In this study, we explored the efficacy of combining IL-10 and the oncolytic Ad-hTERT viroimmunotherapy in Lewis lung carcinoma (LLC) and B16F10-bearing C57BL/6 mice and found that the combination therapy demonstrated a profound antitumor effect. The Ad-hTERT could kill tumor cells directly and increase bulk CD8^+^T cell amount in tumors. Furthermore, Ad-hTERT was available to improve the IFN-γ production of memory CD8^+^T cells in lymph node, spleen and tumors. In addition, IL-10 could also increase CD8^+^T cell amount and enhance the IFN-γ secreted from memory CD8^+^T cells in tumors. The combination therapy showed the most effective anti-tumor effect and stimulation of CD8^+^T cells compared with single treatment. The long-term memory immune response induced by the combination therapy rejected tumor rechallenge. In summary, this study displayed that IL-10 could enhance Ad-hTERT-mediated viroimmunotherapy and CD8^+^T cells are critical for the combination therapy.

## Materials and Methods

### Cell Lines, Viruses, and Reagents

Murine Lewis lung carcinoma (LLC), melanoma B16F10 and HEK293 cell lines were cultured in Dulbecco’s Modified Eagle’s Medium (DMEM) containing 10% fetal bovine serum (FBS) and 1% streptomycin-penicillin (10,000 U/mL) (all: Corning, US) in a humidified incubator at 37°C containing 5% CO_2_. The usage of IL-10 (Murine rIL-10, BD PharMingen) referred to the previous study, and it was injected into tumors at 5 μg/injection (25).

### Oncolytic Ad5-hTERT Virus

For the construction of oncolytic Ad5-hTERT vector, the *E1* region of Ad5 virus was deleted, and hTERT promoter was inserted to promote the expression of *E1*. The oncolytic Ad5-hTERT virus was propagated in HEK293 cells and was purified using cesium chloride (CsCl) density gradient centrifugation. Breifly, 20 ml of purified virus was incubated with 200 ml of proteinase K-SDS solution at 56°C for 2 h, then the viral DNA was precipitated by adding 20 ml of 3 M sodium acetate (pH=5.0) and 600 ml of precooled EtOH (>99.8%; stored at −20°C). Subsequently, the mixture was centrifuged for 8 min at full speed (15,000g) at room temperature and retain the pellet. The pellet was washed by 600 ml of 70% ethanol and then centrifuged at 15,000g at room temperature for 5 min. Dry the pellet in incubator and and resuspend in 25 ml of sterilized dH_2_O. Finally, quantitative PCR (forward primer: 5’-GTTCCACTTGTTGACCGAGC-3’; reverse primer: 5’-CCTAAACGTGTCAACCTT GGA-3’.) was employed to measure the viral particle.

### Cell Viability Assay

The day before treatment, LLC or B16F10 cells were seeded at 60%-70% confluency in 24-well plates. Next day, the cells were incubated with IL-10 (1 ng/well), Ad-hTERT (1,000 viral particles/cell) or IL-10+Ad-hTERT. The cell viability was determined by the CCK-8 assay kit (Sigma-Aldrich) and crystal violet staining. For the CCK-8 assay, the conduction was according to the manuscript of the kit. Briefly, 96 h post-treatment, cells in media were incubated with CCK-8 assay solution for 2 h. Subsequently, the absorbance values were measured at 450 nm using an Infinite F Plex plate reader (TECAN). Triple assays were analyzed.

For the crystal violet staining, the cytopathic effect (CPE) was checked daily until the day 7 post-treatment. Briefly, the cells were fixed with 3.7% formaldehyde and then stained with crystal violet solution for detecting the density of live cell.

### Animal Studies

All animal studies were approved by Animal Welfare and Research Ethics Committee of Capital Medical University. All animal experiments were conducted in accordance with the institutional and national regulations. Female C57BL/6 mice (5 weeks old) were purchased from Capital Medical University Animal Laboratories and were subcutaneously implanted 5 × 10 (6) cells. The injections were conducted as previously described (6). In brief, mice were given intratumoral injections of PBS+vehicle control, IL-10 (5 μg/injection), Ad-hTERT (1 × 10 (8) viral particles/injection), or IL10+Ad-hTERT at 1-day interval for a total of 10 treatments, the materials was injected through needles tracts which were made radially from the tumor center.

To deplete immunocytes in C57BL/6 mice, anti-CD8 (TIB200), anti-CD4 (GK1.5) or anti-NK1.1 (PK136) antibody were intraperitoneally injected every five days from the day before treatments (26). Specific depletion of the respective CD8^+^T, CD4^+^T and NK cells was confirmed by flow cytometric analysis. Tumor sizes were measured every other day using digital calipers, and were calculated using the formula: volume= length × width (2) × π/6.

### Isolation of CD8^+^T Cells

To purify CD8^+^T cells from DLN, the DLN was gently crushed with the back of a syringe plunger and cells passed through a 70 μm filter. To purify CD8^+^T cells from spleen, splenocytes were isolated from mice and erythrocytes were depleted with ACK lysis buffer (Thermo Fisher Scientific, MA, USA). To purify CD8^+^T cells from tumor tissue, the tumor tissue was cut into small pieces (~1 mm (3)) in 200 μl of RPMI 1640 medium containing collagenase type I (0.05 mg/ml; Sigma-Aldrich, USA), collagenase type IV (0.05 mg/ml; Sigma-Aldrich, USA), hyaluronidase (0.025 mg/ml; Sigma-Aldrich, USA), and DNase I (0.01 mg/ml; Roche, Switzerland), then shake at 37°C for 20 min, followed by passing through 70 μm filter. Subseqently, CD8^+^ T cells were purified from the above-mentioned single cell suspension with EasySep™ Mouse CD8^+^ T Cell Isolation Kit (STEMCELL Technologies).

### Flow Cytometric Analysis

The flow cytometric analysis was employed for the analysis of deletion of CD8^+^T, CD4^+^T, and NK cells, and the density of CD8^+^T in tumor tissue suspensions. For the analysis of deletion of CD8^+^T, CD4^+^T and NK cells, the following antibodies were used for staining cells: CD3-Violet 421 (145-2C11), CD4-allophycocyanin (APC) (GK1.5), CD8-PE-Cy7 (53-6.7), NK1.1-PerCP. For analyzing the density of CD8^+^T in tumor tissue suspensions, single-cell suspensions from tumors were prepared, followed by the cell lysis with ACK red blood cell lysis buffer (Lonza) and then antibody staining (CD3-Violet 421, CD4-APC, and CD8-PE-Cy7) at room temperature for 30 min. For analyzing the influence of Ad-hTERT on IL-10R expression on CD8^+^T cells, mouse peripheral CD8^+^T cells were enriched and incubated with Ad-hTERT (Ad-hTERT:CD8^+^T cells = 100:1) for 72 h, then stained with anti-IL-10R (1B1.3a), followed by incubation with rabbit anti-mouse IgG Fc secondary antibody-FITC (ThermoFisher Scientific). After labeling, the cells were fixed in 1% paraformaldehyde, and 10 (5) events were collected and analyzed on a CytoFLEX flow cytometer (Beckman Coulter). Analysis was carried out using the CytExpert software (Beckman Coulter).

### Detection of IFN- γ Production by ELISA

Tumor draining lymph node cells and splenocytes derived CD8+T cells were collected from LLC or B16F10 tumor-bearing C57BL/6 mice, then were stimulated *in vitro* with LLC or B16F10 cells in complete RPMI 1640 medium for 3 days respectively. Cell supernatants were collected for the detection of IFN-γ (interferon-γ) following the manuscript of the IFN-γ ELISA kit (BD PharMingen, San Diego, CA, USA).

### Statistical Analysis

The results are presented as means ± SD. Statistical comparison was analyzed using the two-tailed student t test, one-way ANOVA, or two-way ANOVA. P < 0.05 (*) was considered statistically significant.

## Results

### Combining IL-10 With Oncolytic Ad-hTERT Enhances Antitumor Efficacy

**Figure 1:**
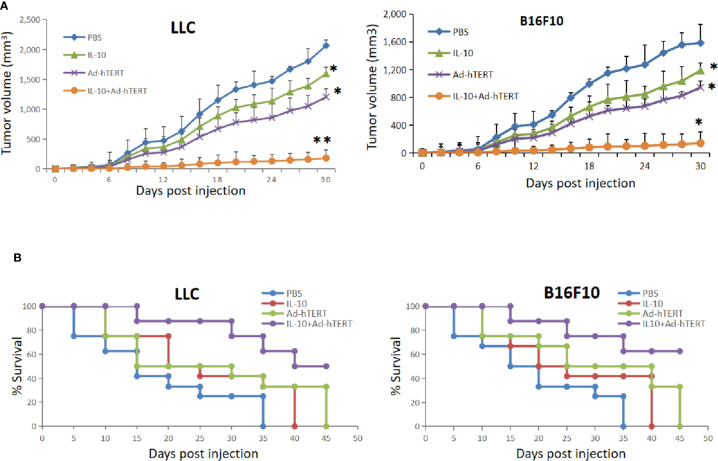
Interleukin 10 (IL-10) enhanced oncolytic Ad-hTERT viroimmunotherapy in Lewis lung carcinoma (LLC) and B16F10 tumor-bearing C57BL/6 mice. **(A)** Average tumor volumes were monitored every other day until the first mouse reached endpoint criteria. Statistical significance was determined with two-way ANOVA tests. **(B)** Kaplan-Meier survival curves demonstrated effects of different treatments. A log rank test was used to determine statistical significance. The figure shows the statistical analysis results of each group compared with the PBS group. N = 12 mice for every group. *p < 0.05. **p < 0.01. Error bar represents SD.

In the context of murine tumor model, we conducted the *in vivo* study to evaluate the effects of IL-10 on Ad-hTERT viroimmunotherapy. Initially C57BL/6 mice were inoculated by subcutaneously injection with LLC or B16F10 cells and then the tumor-bearing mice received treatment. From the day 8 after tumor implant, the mice were intratumorally injected with different amount of IL-10. Compared with other doses, 5 μg/injection of IL-10 showed the strongest effect of inhibiting tumor growth (**Supplementary Figure 1**). Therefore, IL-10 in 5 μg/injection would be combined with Ad-hTERT to treat the tumor-bearing mice. The mice were intratumorally injected with PBS, IL-10, Ad-hTERT, or IL10+Ad-hTERT every other day for 10 treatments. We first assessed the impact of these treatments on tumors with average tumor volume. Both LLC and B16F10 tumor models displayed the consistent results, IL-10 or Ad-hTERT treatment reduced tumor bulks mildly, compared to the negative control (**Figure 1A**). The IL10+Ad-hTERT group, however, leaded to tumor stabilization or regression statistically. Meanwhile, the survival was also monitored to evaluate the effects of these materials on tumor-bearing mice. We found that IL-10 or Ad-hTERT treatment presented a slightly improvement in overall survival compared to the negative control (**Figure 1B**). However, the IL10+Ad-hTERT showed the most efficacious in the treatment regimens, resulting in significantly prolonged overall survival. Taken together, the results indicate that IL-10 and Ad-hTERT possessed antitumor function and the combination therapy had more promising efficacy.

### IL-10 Has No Impact on Ad-hTERT Mediated Oncolysis

**Figure 2:**
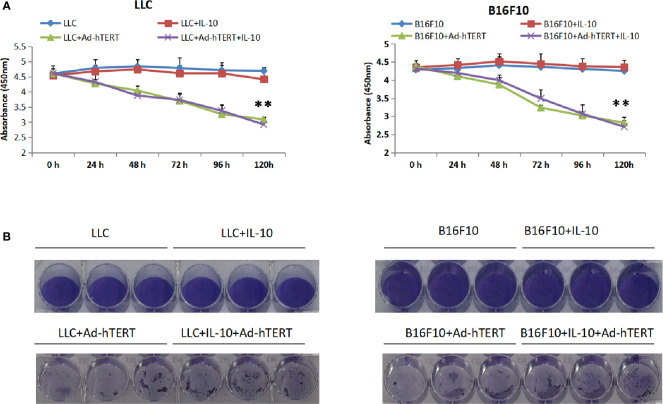
Interleukin 10 (IL-10) showed no cytotoxic effects or enhanced Ad-hTERT mediated oncolysis. **(A)** Cell Counting Kit-8 (CCK-8) viability assay and **(B)** Crystal violet staining assay for Lewis lung carcinoma (LLC) or or B16F10 cells treated with IL-10, Ad-hTERT, or IL-10+Ad-hTERT. CCK-8 viability assay was performed per 24 h and crystal violet staining assay was conducted 120 h post-treatment. Two-tailed t tests were utilized to access statistical significance between different treatment groups. **p < 0.01. Error bar represents SD.

Next, efforts were made to unravel the mechanisms underlying the enhanced efficacy resulting from IL-10. To determine whether IL-10 has direct effect on the Ad-hTERT mediated cytotoxicity of tumor cells, we conducted an *in vitro* Cell Counting Kit-8 (CCK-8) cell viability assay and confirmed the role for IL-10 in the presence or absence of Ad-hTERT infection. After 96 h of treatment, we found that IL-10 treatment alone had mild influence on LLC and B16F10 cell viability. Additionally, the presence of IL-10 did not significantly improve or decrease the effectiveness of Ad-hTERT treatment (**Figure 2A**). We then performed the crystal violet staining 7 days post infection to further detect whether treatment with IL-10 could influence the cell viability and *in vitro* oncolytic capacity by the Ad-hTERT. Results from the two cell lines showed that the addition of IL-10 did not significantly influence the oncolytic of tumor cells by Ad-hTERT (**Figure 2B**, **Supplementary Figure 2**). These results were consistent with the results of the CCK-8 assay.

### The Enhanced Efficacy of IL-10 and Ad-hTERT Combination Therapy Depends on CD8^+^T Cells

Next, we explored the role of immune system, especially the lymphocytes CD8^+^ T, CD4^+^ T and natural killer (NK) cells, in the antitumor effect elicited by the combination therapy. The C57BL/6 mice bearing subcutaneous LLC or B16F10 tumor bulks were intraperitoneally injected with anti-CD8 (TIB210), anti-CD4 (GK1.5) or anti-NK1.1 (PK136) deletion mAbs respectively every 5 days from the day before the viral therapy. Then these C57BL/6 mice were treated with intratumoral injections of PBS, IL-10, Ad-hTERT, or IL10+Ad-hTERT every other day until day 20.

**Figure 3:**
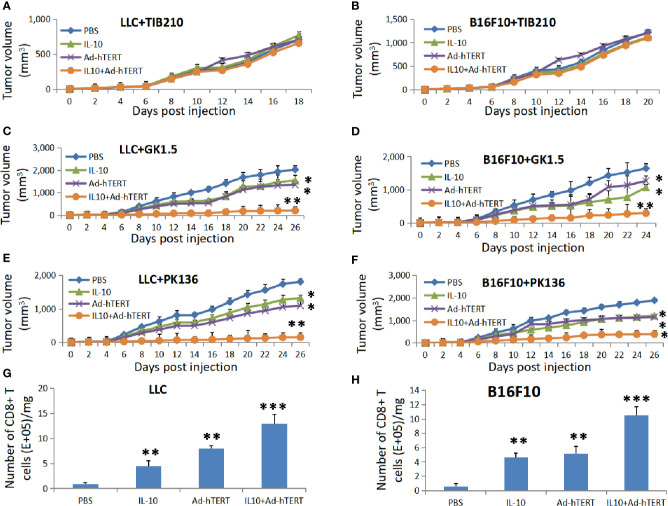
The effects of combination therapy depends on CD8^+^ T Cells. Tumor volumes for the lymphocyte-depleted C57BL/6 mice accepting treatments. **(A, B)** CD8^+^ T, **(C, D)** CD4^+^ T, or **(E, F)** natural killer (NK) cells were depleted from the Lewis lung carcinoma (LLC) or B16F10 tumor-bearing mice. Statistical significance was determined with two-way ANOVA tests. **(G, H)** The CD8^+^T cells density in per milligram (mg) of tumor tissues after IL-10, Ad-hTERT, and IL-10+Ad-hTERT treatments. Statistical significance was determined with one-way ANOVA. N = 12 mice for every group. The figure shows the statistical analysis results of each group compared with the PBS group. *p ≤ 0.05. **p ≤ 0.01. ***p ≤ 0.01. Error bars represent SD.

Flow cytometric analysis of peripheral blood from the mice confirmed specific deletion of CD8^+^ T, CD4^+^ T and NK cells (**Supplementary Figures 3A–C**). As shown in **Figures 3A, B** and **Supplementary Figures 3D, E**, in the CD8^+^ T cells-deleting LLC or B16F10 tumor-bearing mice, all the different treatments resulted in comparable tumor volume and prolong overall survival, indicating that IL-10, Ad-hTERT or IL-10+Ad-hTERT therapies did not affect the tumor development. However, in the context of CD4^+^T (**Figures 3C, D**, **Supplementary Figures 3F, G**) or NK cells (**Figures 3E, F**, **Supplementary Figures 3H, I**) deletion, the LLC or B16F10 tumor-bearing mice processed by IL-10 or Ad-hTERT presented shrinking tumor and improved survival compared with PBS control group. Furthermore, the IL-10+Ad-hTERT remedy showed significantly superior effects in reducing tumor volume and extending overall survival in these mice tumor models. Therefore, these results indicated that CD8^+^T cells play a vital role in the inhibition of tumor development mediated by the IL-10+Ad-hTERT combination treatment.

After determining the critical role CD8^+^T cells in the antitumor effect, we further examined whether IL-10, hTERT or the combination treatment could modulate their density in the tumor microenvironment. For these studies, we established LLC or B16F10 tumor-bearing C57BL/6 mice and treated them as described previously in **Figure 1**. 72 h after the final treatment, these mice were sacrificed and the tumors were collected and subjected to single-cell suspensions for flow cytometric analysis. As shown in **Figures 3G, H**, we observed that IL-10, Ad-hTERT, and IL-10+Ad-hTERT treatments increased the density of CD8^+^T cells in tumor microenvironment, compared to other treatments. It could be concluded that both IL-10 and Ad-hTERT could increase the density of CD8^+^T cells in tumors, and the combination therapy showed better effect on increasing the number of CD8^+^T cells in tumors than single IL-10 or Ad-hTERT treatment.

### Combination Treatment Induced Tumor-Specific Memory CD8^+^ T Cell Responses

**Figure 4:**
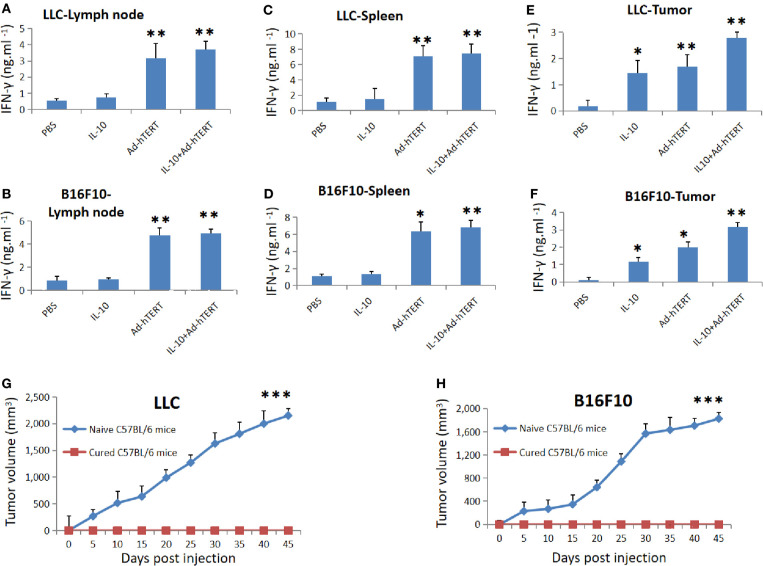
Tumor specific memory immune responses rejected rechallenge. IFN-γ secreted by the CD8^+^ T cells from **(A, B)** lymph node, **(C, D)** spleen, and **(E, F)** tumor tissues was measured 7 days post treatment. Statistical significance was determined with one-way ANOVA. N = 12 mice for every group. The figure shows the statistical analysis results of each group compared with the PBS group. **(G, H)** Average tumor volumes in cured C57BL/6 mice rechallenged by respective LLC (N = 6 mice) or B16F10 (N = 8 mice) cells. Statistical significance was determined with Two-tailed t tests. *p ≤ 0.05. **p ≤ 0.01. ***p ≤ 0.001. Error bars represent SD.

To evaluate whether the intratumoral combination treatment induced specific antitumor CD8^+^ T cells responses, we purified CD8^+^ T cells in tumor draining lymph node (DLN), spleen and tumor tissue in different time points after treatments, and then re-stimulated the CD8^+^ T cells with LLC or B16F10 tumor cells. Considering the key role of IFN-γ in CD8^+^ T cell-mediated immune response, we determined the concentration of IFN-γ secreted by these CD8^+^ T cells 7 days after treatment. We found that the LLC or B16F10 tumor-bearing mice presented the same trend, DLN derived CD8^+^ T cells from Ad-hTERT or IL10+Ad-hTERT-treated mice secreted significantly higher levels of IFN-γ than other groups, and these two groups showed the comparable effect. The IL10+Ad-hTERT induced almost sevenfold or fivefold increase in IFN-γ level compared with the PBS control group in LLC or B16F10 tumor-bearing mice respectively (**Figures 4A, B**). For the IFN-γ produced from spleen derived CD8^+^ T cells, Ad-hTERT or IL10+Ad-hTERT group displayed comparable IFN-γ production, significantly higher levels of IFN-γ than other groups. The IL10+Ad-hTERT dramatically increased to seven or six times higher level of IFN-γ level compared with the PBS control group in LLC or B16F10 tumor-bearing mice respectively (**Figures 4C, D**). In both DLN and spleen derived CD8^+^ T cells, there was no higher level of IFN-γ secreted by CD8^+^ T cell after IL-10 treatment. Meanwhile, we also analyzed the IFN-γ secreted by tumor-resident CD8^+^ T cells after the above mentioned treatments. CD8^+^ T cells from both IL-10 and Ad-hTERT treatment produced more sufficient IFN-γ compared with PBS, and the combination therapy showed the most efficient role through presenting twenty or thirty times higher level of IFN-γ level compared with the PBS control group in LLC or B16F10 tumor-bearing mice respectively (**Figures 4E, F**). Considering the complexity of IL-10-based therapy, we incubated various concentrations of IL-10 with mouse peripheral CD8^+^ T cells for 72 h, and then evaluated the viability of CD8+ T cells. We found that intermediate level of IL-10 could improve the viability of CD8^+^ T cells, while high level of IL-10 resulted in reduced viability of CD8^+^ T cells (**Supplementary Figure 4**). In addition, the Ad-hTERT had no influence on IL-10R expression on CD8^+^T cells (**Supplementary Figure 5**). Taken together, both IL-10 and Ad-hTERT could induce specific tumor-resident CD8^+^ T cells responses, and the combination therapy showed stronger effects.

To determine whether the IL-10+Ad-hTERT combination therapy induced memory antitumor CD8^+^ T cell responses, we selected the combination therapy-cured mice, and rechallenged them through injection of respective tumor cell lines in contrary side to the first inoculation. We found that the rechallenged tumors couldn´t grow in all the mice, but both LLC and B16F10 tumors grew progressively in naive mice (**Figures 4G, H**). These results indicate that the IL-10+Ad-hTERT combination therapy-induced tumor regression produced memory tumor-specific immunity capable of protecting the cured mice from rechallenge.

## Discussion

Oncolytic viruses represent a potential alternative for tumor treatment and the efficacy of oncolytic viroimmunotherapy benefits from both the direct oncolysis and the effective activation of antitumor immunity in tumor microenvironment (27). The ability of enabling the T cell-mediated immune responses to clear residual tumor bulks even outweighs the oncolysis in oncolytic adenovirus mediated antitumor efficacy (6). However, monotherapy with oncolytic adenoviruses showed disappointing outcome in clinical trials and the possible reasons might be their failure to activate antitumor immune response.

In this work, we demonstrated that localized IL-10 could enhance oncolytic adenovirus viroimmunotherapy in immunocompetent murine tumor models. Our results support the speculation that the immunity in tumor microenvironment could be activated to strengthen virus-mediated immunological efficacy. Both oncolytic adenovirus and IL-10 were required for efficient therapeutic activity, because single Ad-hTERT or IL-10 treatment showed only mild efficacy while the additive exhibited significantly improved prognosis. The role of IL-10 in combination therapy in our study is consistent with the study in which IL-10 is armed in an oncolytic vaccinia virus for treating pancreatic cancer (28). The oncolytic vaccinia virus armed with IL-10 treatment contributed to significantly reduced frequencies of antiviral T cells, whereas the frequency of tumor-specific CD8^+^ T cells was comparable to oncolytic vaccinia virus monotherapy at days 8 and 24 post-infusion. At day 16 post-infusion, the increased frequency of tumor-specific CD8^+^ T cells was detected. In our study we found that both IL-10 and Ad-hTERT showed potential in increasing the amounts of CD8^+^ T cells in tumors and improving specific anti-tumor CD8^+^ T cells responses. Therefore, their combination further enhanced the effect of CD8^+^ T cell on repressing tumors. It is likely that IL-10 is a chemoattractant for CD8^+^ T cells (29) and the oncolytic Ad5-hTERT could improve the production of CD8^+^ T cells in the tumor microenvironment (30). These findings support that combining IL-10 and Ad-hTERT has an effect on increasing the number of tumor residual CD8^+^ T cells.

Monocytes and lymphocytes are the primary source of IL-10, and reports on the role of IL-10 in tumor progression are controversial. It was found that large amount of IL-10 secreted by tumor-associated macrophages (TAMs) contributed to breast cancer drug resistance (31). Among other effects, the adoptive transfer of IL-10-expressing Treg cells into Rag2^−/−^ mice inhibits colorectal carcinomas, this model reflected the suppression of host innate inflammatory response by IL-10 was pivotal in interrupting carcinogenesis (26, 32). It is also reported that IL-10-deficient murine models developed to bacteria-induced carcinogenesis at high rates (33). And IL-10 could also enhance NK lysis of tumor cells through downregulating the expression of inhibitory ligand, major histocompatibility complex class I molecule (MHCI), on tumor cells (34). Although the combination therapy in our study ultimately resulted in prolonged survival in the murine tumor models, we shouldn’t neglect the contradictory role of IL-10 in CD8^+^T cells. The different direct roles of IL-10 on CD8+T cells are associated with resident physiopathological states. Although the lack of IL-10 signaling has no impact on memory CD8^+^T cell development following vaccination (35), IL-10 reduces CD8^+^ T cell antigen sensitivity and capacity to control pathogen burden in chronic viral infection (36). Meanwhile, IL-10 shows stimulating roles in tumor-resident CD8^+^ T cell through expanding cytotoxicity of CD8^+^ T cells and leading to an increase in the expression of IFN-γ in CD8^+^ T cells, and the inhibition of T-cell trafficking from lymphoid organs does not impair IL-10-induced activation of tumor-resident CD8^+^ T cells (24).

We could detect IFN-γ production of CD8^+^ T cells in tumors after IL-10 or hTERT treatment by re-stimulated CD8^+^ T cells with LLC or B16F10 cells, and CD8^+^ T cells from draining lymph node (DLN) and spleen post hTERT treatment could also secrete IFN-γ after re-stimulation. Therefore, combining IL-10 and hTERT could generate more tumor specific CD8^+^ T cells in more organs than single treatment.

The analysis of results from lymphocytes-depleted C57BL/6 mice indicates that CD8^+^ T cells-mediated immune response plays a key role in antitumor efficacy in immunocompetent hosts. Although NK cells also play important roles in inhibiting tumors (37–39), it is widely accepted that adaptive immunity are more important antitumor tools. T cells play a pivotal role in oncolytic virus-mediated tumor regression in immunocompetent hosts. Li et al. explored the effects of T cells in oncolytic adenovirus therapy by depleting T cells in hamsters using an anti-Syrian hamster CD3 mAb (6). They found that oncolytic adenovirus induced strong virus-specific and tumor-specific T-cell responses and the depletion of T cells prolonged the persistence of adenovirus, but also eliminated the antitumor efficacy. Therefore, to further improve the combination therapy through decreasing the virus-specific T-cell response and increasing tumor-specific T-cell response, we should next evaluate the effect of the sequential therapy and show the most promising antitumor efficiency.

Taken together, our study proves the antitumor role of IL-10 in our mouse models and indicates that combining IL-10 with Ad-hTERT provides a potential antitumor candidate, CD8^+^ T cells are critical for the combination therapy efficacy.

## Data Availability Statement

The original contributions presented in the study are included in the article/**Supplementary Material**. Further inquiries can be directed to the corresponding authors.

## Ethics Statement

The animal study was reviewed and approved by Animal Welfare and Research Ethics Committee of Capital Medical University.

## Author Contributions

DC designed the research, conducted experiments, analyzed the data, and wrote the manuscript. LH performed *in vitro* experiments and analyzed data. HZ and YZ designed the research and revised the manuscript. All authors contributed to the article and approved the submitted version.

## Funding

This work was supported by National Natural Science Foundation of China (No. 81802263).

## Conflict of Interest

The authors declare that the research was conducted in the absence of any commercial or financial relationships that could be construed as a potential conflict of interest.
