# Supplementary material for: Combining IL-10 and Oncolytic Adenovirus Demonstrates Enhanced Antitumor Efficacy Through CD8+ T Cells
Source: Front Immunol. 2021 Feb 26;12:615089. doi: 10.3389/fimmu.2021.615089 (PMC7952747; doi:10.3389/fimmu.2021.615089)
Supplement: Supplementary file 1 [file DataSheet_1.docx]

Supplementary Material

**Supplementary Figure 1. The influence of diverse concentrations of IL-10 on the growth of lung tumor.** The LLC and B16F10 based mouse lung tumor bulks were injected with different amounts of IL-10, then the tumor volume was measured per two days. N = 12 mice for every group.

**Supplementary Figure 2. Crystal violet staining assay for LLC and B16F10 cells treated with IL-10, Ad-hTERT or IL-10+Ad-hTERT.** Crystal violet staining assay was performed 120 hours post-treatment and was quantified. Two-tailed t tests were utilized to access statistical significance between different treatment groups. **, *p* < 0.01. Error bar represents SD.

**Supplementary Figure 3. The antitumor efficacy of combination therapy is influenced by CD8^+^ T Cells.** (A-C) Confirmation of depletion of CD8+ T, CD4+ T and NK cells in mice. Representative flow cytometric analysis of tumors isolated from mouse treated with CD8^+^ T, CD4^+^ T and NK cell-depleting antibodies. Numerical values represent percentage of (A) CD4^+^ or (B) CD8^+^ T cells out of CD3^+^expressing cells, or the (C) NK1.1^+^ cells. (D-I) Kaplan-Meier survival curves demonstrated effects of different treatments. LLC or B16F10 cells were injected treated withA log rank test was used to determine statistical significance. N = 12 mice for every group.

**Supplementary Figure 4.** The viability of CD8^+^ T cells, following 72-h incubation with various concentrations of IL-10.Statistical significance was determined with one-way ANOVA.The figure shows the statistical analysis results of each group compared with the negative group (IL-10 concentration=0).*, *p* ≤ 0.05. Error bars represent SD.

**Supplementary Figure 5.** The influence of Ad-hTERT on IL-10R expression on CD8^+^ T cells . CD8^+^T cells group i the negativ control, and in CD8^+^T cells +Ad-hTERT goup, CD8^+^T cells were incubated with Ad-hTERT, and then the IL-10R level was measured by flow cytometry.
